# Diagnostic performance of magnetic resonance imaging for colorectal liver metastasis: A systematic review and meta-analysis

**DOI:** 10.1038/s41598-020-58855-1

**Published:** 2020-02-06

**Authors:** Yitao Mao, Bin Chen, Haofan Wang, Youming Zhang, Xiaoping Yi, Weihua Liao, Luqing Zhao

**Affiliations:** 1 Department of Radiology, Xiangya Hospital, Central South University, Changsha, Hunan 410008 China; 2 Department of Interventional Radiology, The First Affiliated Hospital, Sun Yat-sen University, Guangzhou, Guangdong 510080 China; 3 Department of Interventional Radiology, The Third Affiliated Hospital, Sun Yat-sen University, Guangzhou, Guangdong 510630 China; 4 Department of Pathology, Xiangya Hospital, Central South University, Changsha, Hunan 410008 China; 5 Department of Pathology, School of Basic Medical Science, Xiangya School of Medicine, Central South University, Changsha, Hunan 410013 China; 6 Department of Pathology and State Key Laboratory of Liver Research, The University of Hong Kong, Hong Kong, China

**Keywords:** Cancer imaging, Cancer screening

## Abstract

The prognosis of colorectal cancer (CRC) is largely dependent on the early detection of hepatic metastases. With the advantages of nonradioactivity and the availability of multiple scanning sequences, the efficacy of magnetic resonance imaging (MRI) in the detection of colorectal liver metastases (CRLM) is not yet clear. We performed this meta-analysis to address this issue. PubMed, Embase, and the Cochrane Library were searched for studies reporting diagnostic performance of MRI for CRLM. Descriptive and quantitative data were extracted. The study quality was evaluated for the identified studies and a random effects model was used to determine the integrated diagnosis estimation. Meta-regression and subgroup analyses were implemented to investigate the potential contributors to heterogeneity. As a result, seventeen studies were included for analysis (from the year 1996 to 2018), comprising 1121 patients with a total of 3279 liver lesions. The pooled sensitivity, specificity, and diagnostic odds ratio were 0.90 (95% confidence intervals (CI): 0.81–0.95), 0.88 (0.80–0.92), and 62.19 (23.71–163.13), respectively. The overall weighted area under the curve was 0.94 (0.92–0.96). Using two or more imaging planes and a quantitative/semiquantitative interpretation method showed higher diagnostic performance, although only the latter demonstrated statistical significance (*P* < 0.05). Advanced scanning sequences with DWI and liver-specific contrast media tended to increase the sensitivity for CRLM detection. We therefore concluded that contemporary MRI has high sensitivity and specificity for screening CRLM, especially for those with advanced scanning sequences. Using two or more imaging planes and adopting a quantitative/semiquantitative imaging interpretation may further improve diagnosis. However, the MRI results should be interpreted with caution because of substantial heterogeneity among studies.

## Introduction

Colorectal cancer (CRC) is one of the most common malignancies in the world. In recent decades, the mortality rate of CRC has decreased due to the development of diagnostic techniques and optimization of treatment strategies, including surgery, chemotherapy and palliative therapies^1^. However, the incidence of CRC is increasing in the general population, especially in those younger than 40 years old^2^. The prognosis of CRC patients is largely dependent on local tumor staging and the presence of distant metastasis. In those metastatic cases, the liver is the most frequently involved organ, followed by the lung^3,4^. Almost one out of five patients present with liver metastases (LM) at the diagnosis of CRC^5^, and up to 50% of patients manifests LM at some time during the disease course^6^.

Detection of colorectal liver metastases (CRLM) at an early stage is crucial for improving survival because it facilitates the selection of potential patients who will benefit from curable liver surgery and avoidance of those who are not appropriate surgery candidates^7^. The current diagnostic methods used for the evaluation of CRLM are heterogeneous. Among various imaging methods, such as computed tomography (CT), positron emission tomography (PET) combined with CT and ultrasonography (US), magnetic resonance imaging (MRI) has its superiority in LM detection due to its superior soft tissue resolution, multiple scan sequences, innovative MRI techniques, and the use of hepatocyte-specific contrast agents^8^. Recent studies have shown that liver MRI performed excellently in determining CRLM with both high sensitivity and specificity^9–11^. However, controversy exists regarding the role of MRI scanning and whether it can replace other imaging methods in the diagnosis of CRLM.

Therefore, we undertook this meta-analysis to evaluate the possible benefit of contemporary MRI to differentiate metastatic liver lesions from nonmetastatic liver lesions in patients with CRC.

## Materials and Methods

### Search strategy

A comprehensive online literature search was performed for studies evaluating MRI for screening hepatic metastases in CRC patients. We searched PubMed, EMBASE, and the Cochrane Library (the search was last updated on Feb 20, 2019) with a search algorithm based on a combination of the following five factors: (1) colorectal cancer, (2) liver, (3) metastasis, (4) MRI, and (5) diagnosis. The synonyms of the five factors were searched as follows: ([“carcinoma of colon”] OR [“colon carcinoma”] OR [“colon cancer”] OR [“colonic cancer”] OR [“colonic neoplasm*”] OR [“colorectal cancer”] OR [“colorectal neoplasm*”] OR [“rectal cancer”] OR [“rectal carcinoma”] OR [“rectal neoplasm*”] OR [“rectum cancer”] OR [“rectum neoplasm*”] OR [“rectum carcinoma”]) AND ([“liver”] OR [“hepatic”]) AND ([“metastatic”] OR [“metastas*”]) AND ([“magnetic resonance”] OR [“MRI”] OR [“MR”]) AND ([“diagnosis”] OR [“diagnostic”] OR [“accuracy”] OR [“performance] OR [“detection”] OR [“detectability”] OR [“sensitivity”] OR [“specificity”]). Bibliographies of the retrieved articles were carefully screened for potentially relevant studies. The search was restricted to “humans” and the “English” language.

### Study selection

Studies were included when the following criteria were met: (1) patients diagnosed with CRC; (2) MRI used as the evaluation tool for detection of hepatic metastasis; (3) histopathology (surgery, biopsy),or intraoperative ultrasonography/manual palpation, or clinical/imaging follow-up used as the reference standard for comparison; (4) sufficient data provided to reconstruct 2 × 2 tables of true positives (TP), false positives (FP), false negatives (FN), and true negatives (TN); (4) studies based on a per-lesion analysis; (5) the number of patients was no less than 10; (6) English was the publication language; and (7) original article was the publication type. When there were replicated data presented in different studies, only the study with the largest sample size (i.e., the number of patients) was included. Only full-length articles were included. Case reports, reviews, conference abstracts, and letters, as well as papers using animal models were excluded. In some cases, MRI was used for the evaluation of hepatic metastasis in CRC, but focused on treatment response rather than on diagnostic performance, these articles were also excluded.

### Data extraction and quality assessment

Data from the eligible studies were extracted independently by two of the authors (Mao and Zhao), and a third author (Liao) resolved any disagreement pertaining to the extraction of data, and the final consensus was made via discussion. For each report, the relevant information was extracted, including the name of first author, journal, country of origin, year of publication, studied population, study design (prospective or retrospective), patient enrollment procedure, scanner type, type of machine, magnetic field strength, scanning sequences, type of contrast agent (CA) used, number of imaging planes, minimum slice thickness, imaging interpretation method of positive MRI test, and reference standard. Values for TP, FP, FN, and TN findings for the MRI test were also recorded from each study.To assess the methodological quality and applicability of the included studies, the Quality Assessment of Diagnostic Accuracy Studies-2 (QUADAS-2) tool was used^12^. Each eligible study was evaluated by two of the authors (Mao and Zhao) independently, and discrepancies were resolved by discussion with a third author (Liao).

### Statistical analysis

We implemented all analyses on a per-lesion data basis. Based on the 2 × 2 tables, a bivariate model was used to obtain the weighted summary estimates of sensitivity and specificity, which were the main outcome measures, and a hierarchical summary receiver operating characteristic (HSROC) model was used to establish summary receiver operating characteristic (SROC) curves with 95% confidence intervals (CI) and prediction regions^13–15^. The pooled positive likelihood ratio (PLR) and negative likelihood ratio (NLR), as well as diagnostic odds ratio (DOR), which is a metric that integrates both sensitivity and specificity in its calculation^16^, for MRI detection of CRLM were also calculated. When several MRI sequences were separately evaluated for CRLM detection, the results of the most advanced sequence or most comprehensive protocol were selected for analysis. The data from each study were pooled by a fixed or random effects model based on the degree of heterogeneity. Heterogeneity across the included studies was assessed by Cochran’s *Q* test and Higgins *I*^2^ test. Substantial heterogeneity was considered present when *P* < 0.05 for Cochran’s *Q* test or *I*^2^ > 50%^17^. Publication bias was assessed by visual judgment of the Deeks’ funnel plot and the *P* value derived from Deeks’ asymmetry test^18^. In addition, meta-regression analysis, with a test standard of *α* = 0.10, was performed to explore the possible sources of heterogeneity among individual studies on pooled diagnostic performance, followed by subsequent subgroup analysis for those suggested variables.

All analyses were conducted using Stata version 13.0 (StataCorp, College Station, TX), with *P* < 0.05 being considered statistically significant.

### Ethical approval

This study is a meta-analysis on the published literatures and does not belong to studies with experimental human or animal subjects. And it should be exempted from the requirement of obtaining informed consents since the manuscript does not include any potentially identifiable patient images or data, or other identifiable information about individual patients.

## Results

### Selected studies

**Figure 1:**
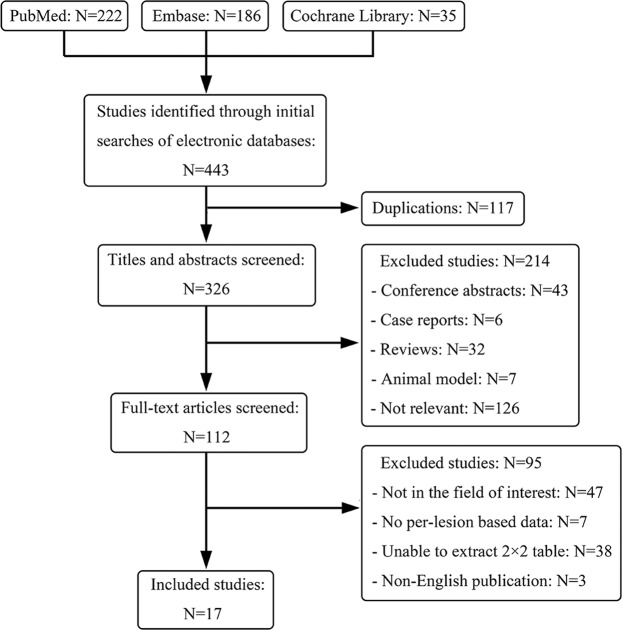
Flow chart of studies identified, excluded and included.

The initial search identified 443 potential articles, and 117 out of 443 were excluded due to duplicated articles. A total of 214 articles were excluded by reviewing the titles and abstracts. Full-text reviews were conducted on the remaining 112 articles, and 95 studies were rejected. Ultimately, 17 studies evaluating the diagnostic performance of MRI for LM detection in patients with CRC were considered for further analyses. As a whole, 1121 patients with a total of 3279 liver lesions were included in this meta-analysis. The screening process of the identified articles and reasons for exclusion are shown in Fig. 1. The size of the study population varied from 15 to 184 patients, with the total number of liver lesions ranging from 37 to 533. Of all studies, three claimed that they were prospectively designed^11,19,20^, eleven were retrospectively designed^9,10,21–29^, and the remaining were unclear^30–32^. The principal characteristics of the included studies are summarized in Table 1 and Table 2.

**Table 1:** Characteristics of the included studies.

Study & Year	Nation	No. of patient (M/F)	Age (yr),mean (range)	Patient enrollment	No. of lesion	Study design	Blind	Analysis method	Reference standard
Brendle (2016)	Germany	15 (9/6)	45 (10–62)	C	37	Retro	Y	Qualitative	H/IF
Cantwell (2008)	USA	33 (22/11)	63	NC	110	Retro	Y	Semiquantitative	H/IF
Chiaradia (2014)	France	15 (8/7)	64 (38–88)	C	79	Retro	Y	Quantitative & Qualitative	H
Cho (2015)	Korea	65 (35/30)	66.6	NR	121	Retro	NR	Qualitative	H/IF
Colagrande (2016)	Italy	54 (35/19)	69.5 (47–75)	NC	135	Retro	Y	Qualitative	IOUS & H
Hwang (2018)	Korea	175 (115/60)	59.3	C	474	Retro	Y	Semiquantitative	H
Kartalis (2011)	Sweden	15 (7/8)	64 ± 8	NR	40	Retro	Y	Qualitative	H/IF
Kong (2008)	UK	65 (42/23)	65*	NR	171	Retro	Y	NR	H/IF
Mainenti (2010)	Italy	34 (20/14)	63 (29–81)	C	57	Retro	Y	Qualitative	BP/IOUS/H/IF
Oba (2018)	Japan	59 (42/17)	59 (30–81)	C	275	Retro	Y	NR	H/IF
Rappeport (2007)	Denmark	35 (16/19)	62* (33–74)	C	124	Pro	Y	Qualitative	H/IF/IOUS
Rojas (2014)	Italy	51 (31/20)	65* (28–79)	NC	156	Retro	NR	Qualitative	H
Said (2000)	USA	19 (12/7)	53 (32–73)	NR	39	NR	Y	Qualitative	H
Schulz (2016)	Norway	46 (29/17)	67* (33–85)	NR	336	Pro	Y	Qualitative	H/IF
Shiozawa (2017)	Japan	69 (46/23)	66 (34–86)	NR	133	NR	Y	Semiquantitative	H/IF
Sivesgaard (2018)	Denmark	80 (47/33)	68* (29–86)	C	533	Pro	Y	Semiquantitative	H/IF/IOUS/CE-CT
Zerhouni (1996)	USA	166(NR)	NR	NR	459	NR	Y	Semiquantitative	H/IF

*Median age; BP: bimanual palpation; C: consecutive; CE-CT: contrast-enhanced computed tomography; H: histopathology; IF: imaging follow–up; IOUS: intraoperative ultrasonography; NC: non-consecutive; NR: not reported; Pro: prospective; Retro: retrospective; Y: yes.

**Table 2:** Descriptions of MR scanning in included studies.

Study & Year	Field Strengthen (T)	Sequences	Scanner type	Machine	Contrast agent	No. of imaging planes	MST (mm)
Brendle (2016)	3	T2WI/STIR/DWI	Siemens	Biograph mMR	none	2	5
Cantwell (2008)	1.5	T1WI/T2WI/DCE-MRI	GE/Siemens	Signa Advantage/Magnetom	Gd-related	2	5
Chiaradia (2014)	1.5	T1WI/T2WI/IVIM-DWI/DCE-MRI	Siemens	Avanto	Gd-related	3	3
Cho (2015)	1.5 or 3	T1WI/T2WI/DWI/DCE-MRI	Philips	Intera Achieva	Gd-related	2	2
Colagrande (2016)	1.5	T1WI/T2WI/DWI/DCE-MRI	Philips	Achieva	Gd-related	1	4
Hwang (2018)	3	T1WI/T2WI/DWI/DCE-MRI	Philips	InteraAchieva	Gd-related	3	NR
Kartalis (2011)	1.5	T1WI/T2WI/DCE-MRI	Siemens	Magnetom Avanto	Gd-related	3	1.5
Kong (2008)	1.5	T1WI/T2WI/CE-MRI	Philips	Gyroscan Intera Master	MnDPDP	2	7
Mainenti (2010)	1.5	T1WI/T2WI/DCE-MRI	Philips	Gyroscan Intera	Gd-related/SPIO	2	4
Oba (2018)	NR	DCE-MRI	NR	NR	Gd-related	NR	NR
Rappeport (2007)	1.5	T1WI/T2WI/DCE-MRI	GE	Horizon Signa LX	SPIO	2	6
Rojas (2014)	1.5	DCE-MRI	GE	Signa	Gd-related	1	2
Said (2000)	0.5/1.5	T1WI/T2WI/STIR/DCE-MRI	Picker/GE	Vista Hi Q/Signa	SPIO	1	7
Schulz (2016)	1.5	T1WI/T2WI/DWI/SPIR/DCE-MRI	Philips	Achieva	Gd-related	2	4
Shiozawa (2017)	1.5	T1WI/T2WI/DWI/DCE-MRI	GE	Signa Lx	Gd-related	2	NR
Sivesgaard (2018)	1.5	T2WI/DWI/PDFF/DCE-MRI	Philips	Ingenia	Gd-related	2	4
Zerhouni (1996)	1.0/1.5	T1WI/T2WI/STIR	NR	NR	none	1	10

DCE: dynamic contrast-enhanced; DWI: diffusion-weighted imaging; Gd: Gadolinium; GE: General Electric Company; MnDPDP: manganese dipyridoxyl diphosphate; MRI: magnetic resonance imaging; NR: not reported; PDFF: proton density fat fraction; SPIO: superparamagnetic iron oxide; SPIR: spectral presaturation with inversion recovery; MST: minimum slice thickness; STIR: short time inversion recovery; T1WI: T1-weighted imaging; T2WI: T2-weighted imaging.

### Quality assessment and evaluation of publication bias

**Figure 2:**
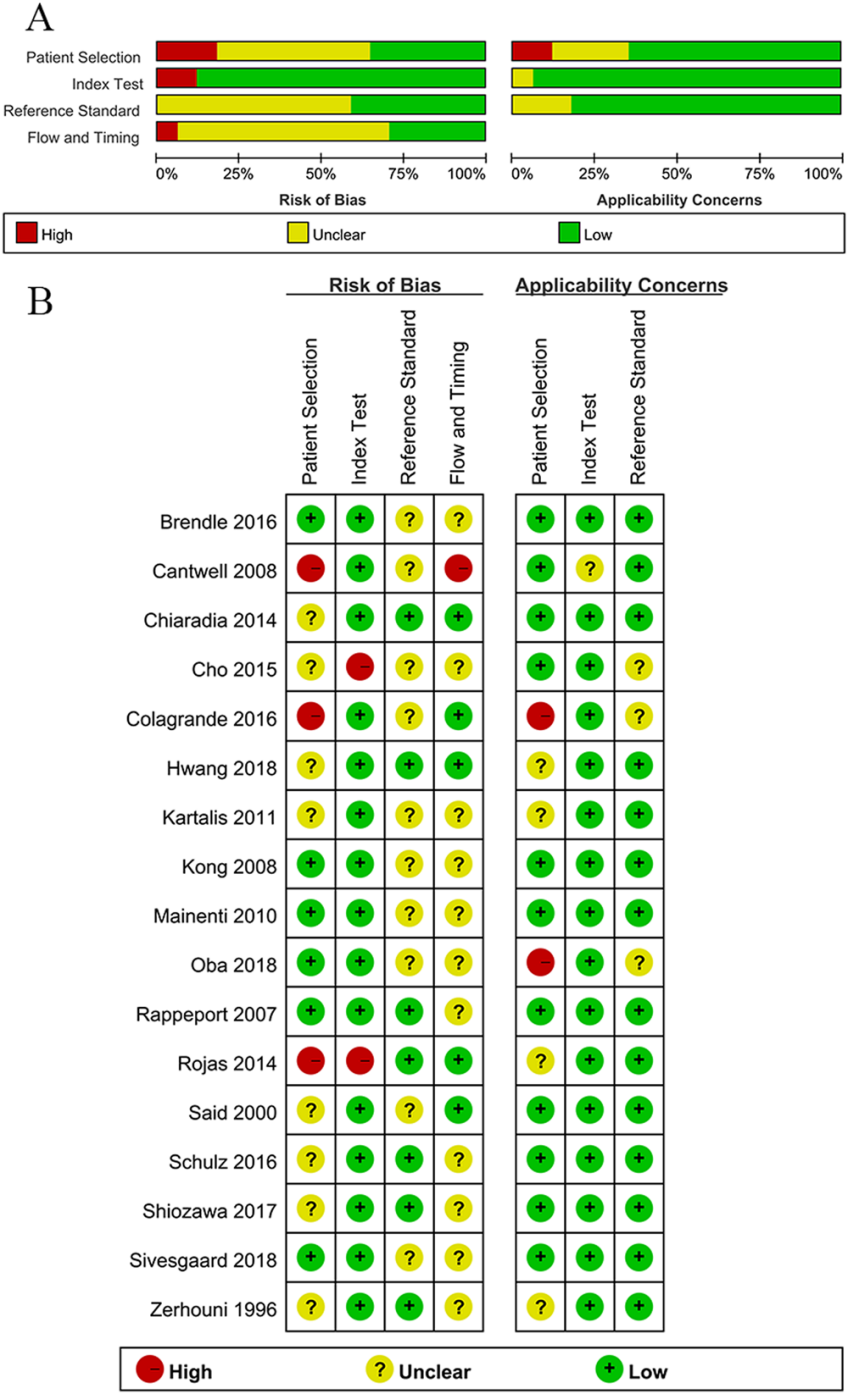
Methodological quality of the included 17 studies using assessment tool of QUADAS-2. QUADAS-2 = Quality Assessment of Diagnostic Accuracy Studies-2. (**A**) Grouped bar charts of risk of bias (left) and concerns for applicability (right). (**B**) Quality assessment for each individual study.

**Figure 3:**
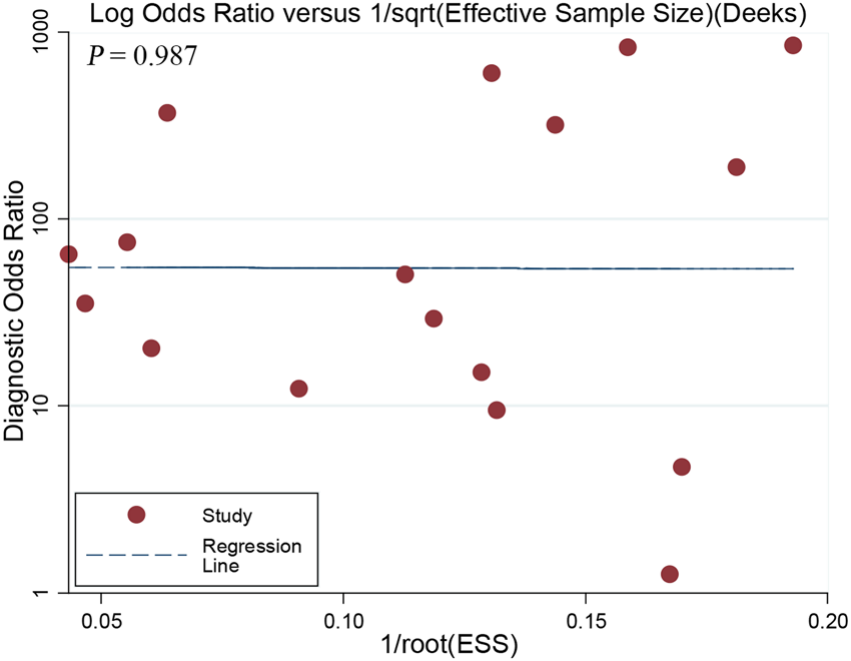
Deeks *et al*.'s funnel plot for per-lesion analysis. A *P* value of 0.987 suggests that no publication bias was demonstrated. ESS = effective sample size.

The quality of the 17 included articles according to the QUADAS-2 assessment tool was considered moderate (see Fig. 2). Ten out of the 17 studies satisfied at least five of the seven QUADAS-2 domains and were considered high quality. For the patient selection domain, three studies were considered to possess a high risk of bias due to nonconsecutive enrollment procedures^22,24,29^. Two studies were considered to have high concern for applicability; one study only included patients with histologically uniform primary tumor and with oligometastasis (<5 metastases)^24^, and one study addressed only those LM that responded to preoperative chemotherapy^28^. For the index test domain, there was a high risk of bias in two studies, as it was not clear whether the interpretation of MRI was blinded to the reference standard^9,29^. None of the studies were considered to have high concern for applicability. For the reference standard domain, there was no high risk of bias or high concern for applicability in all studies. For the flow and timing domain, one study was considered to have a high risk of bias as different reference standards were adopted within the study, and the interval between MRI scan and reference standard was unclear^22^. Note that a majority of the included studies did not use a single reference standard within the study because of the limited feasibility for histologically verifying all hepatic lesions. Deeks’ funnel plot asymmetry test^18^ was used to evaluate the publication bias, as shown in Fig. 3. The slope was flat, and no publication bias was found (*P* = 0.987).

### Diagnostic accuracy of MRI in detecting CRLM

**Figure 4:**
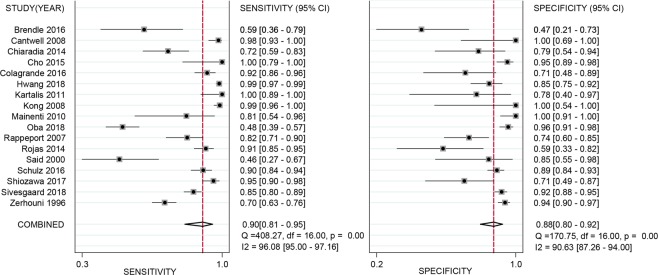
Forest plots of the sensitivity, specificity with corresponding 95% CIs for MRI imaging in detection of liver metastases in patients with colorectal cancer.

**Figure 5:**
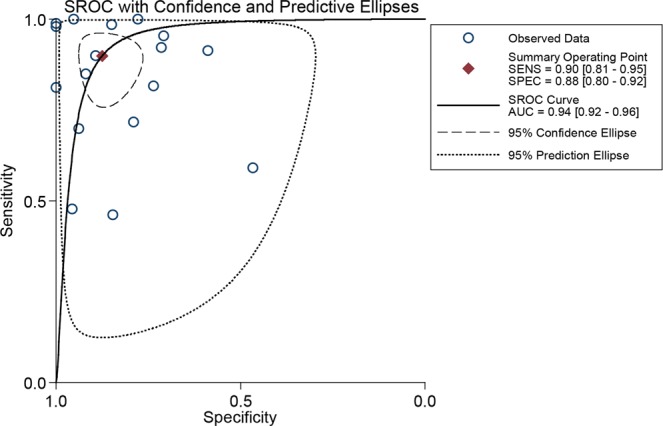
Summary receiver operating characteristic (SROC) curve of the diagnostic performance of MRI for detection of liver metastases in patients with colorectal cancer.

The pooled sensitivity and specificity of all 17 studies for MRI to detect hepatic metastases in patients with CRC were calculated based on the random effects method since significant statistical heterogeneity did exist (*I*^2^ = 96.1% for sensitivity; *I*^2^ = 90.6% for specificity). As shown in Fig. 4, the pooled sensitivity and specificity were 0.90 (95% CI: 0.81–0.95) and 0.88 (95% CI: 0.80–0.92), respectively. Additionally, the DOR, PLR, and NLR were 62.19 (95% CI: 23.71–163.13), 7.21 (95% CI: 4.38–11.86), and 0.12 (95% CI: 0.06–0.23), respectively. The SROC curve of MRI for the diagnosis of CRLM was calculated by sensitivity against specificity (Fig. 5). The curve represented the overall test performance of all included studies. The curve showed that the 95% confidence and prediction regions displayed large variances among studies, further indicating that substantial heterogeneity existed among the studies. The overall weighted area under the SROC curve (AUC) was 0.94 (95% CI: 0.92–0.96).

### Meta-regression and subgroup analysis

**Figure 6:**
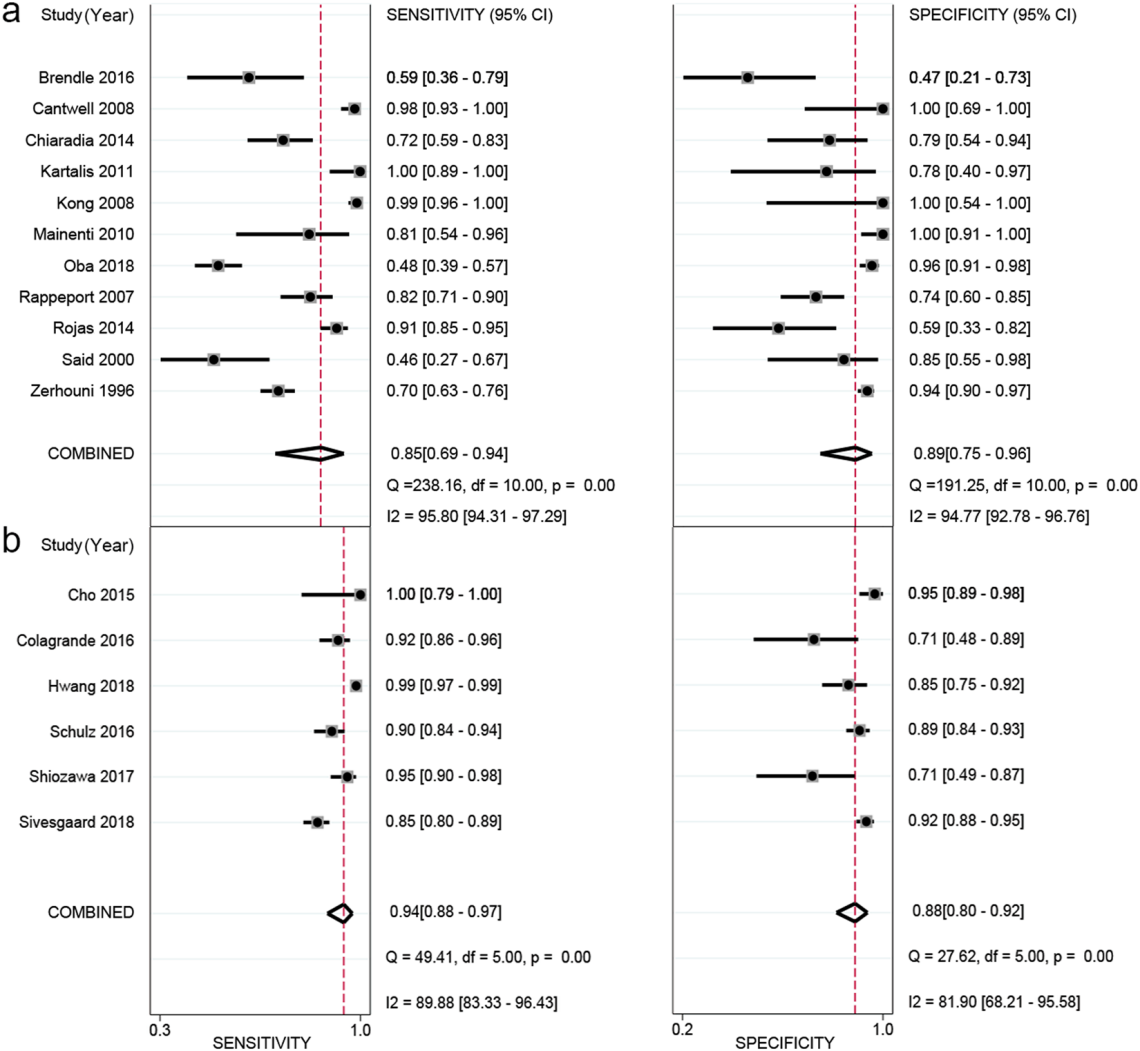
Subgroup forest plots of the sensitivity, specificity with corresponding 95% CIs for MRI imaging in detection of liver metastases in patients with colorectal cancer. (**a**) The studies which do not include both DWI and hepatocellular phase images; (**b**) the studies which include both DWI and hepatocellular phase images.

**Figure 7:**
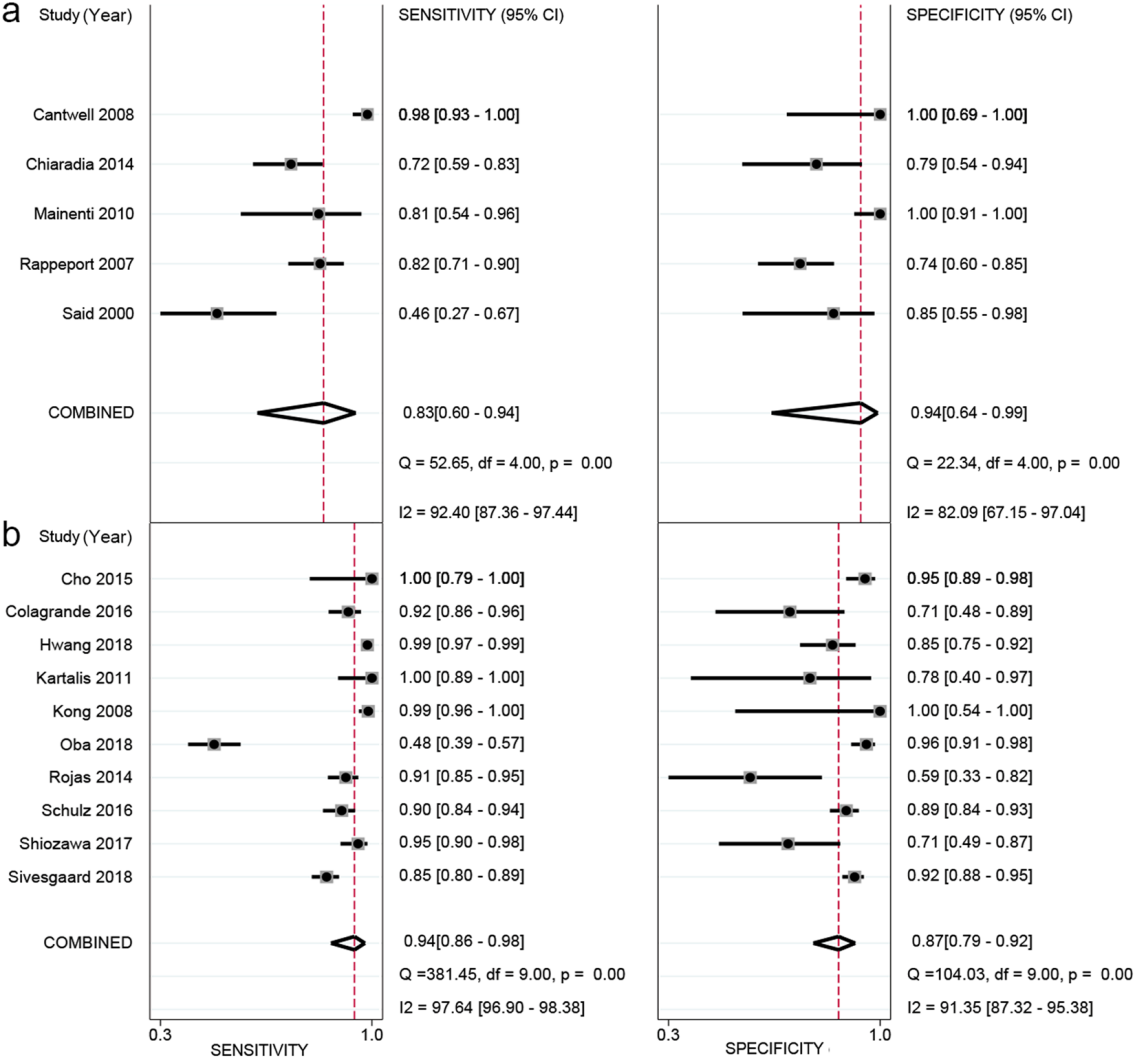
Subgroup forest plots of the sensitivity, specificity with corresponding 95% CIs for MRI imaging in detection of liver metastases in patients with colorectal cancer. (**a**) the studies which do not use liver-specific contrast media as contrast agents; (**b**) the studies which use liver-specific contrast media as contrast agents.

As indicated by the meta-regression analysis, a majority of covariates, including publication year, study design, patient enrollment procedure, magnetic field strength, scanning sequences, minimum slice thickness, lesion size, and reference standard, were not strongly associated with accuracy. The factors that potentially showed significant contributions to the heterogeneity, at a test standard of *α* = 0.10, were number of imaging planes, whether contrast enhancement was used, type of CA, region, and imaging interpretation method (Table 3). Subsequent subgroup analyses were performed for the above-identified variables by meta-regression whose sample size was no less than four studies. The subgroup of no contrast enhancement and type of CA using superparamagnetic iron oxide (SPIO) were excluded from the subgroup analysis because these two subgroups had small sample sizes (i.e., the subgroups included 2 and 3 studies, respectively), making the result unstable. Compared to the respective subgroup comparisons, the results indicated that the use of 2 or more imaging planes, studies from East Asia, and imaging interpreted with quantitative/semiquantitative methods demonstrated higher diagnostic performance for pooled sensitivity or specificity, although significant statistics were found only in the between-subgroup comparison of imaging interpretation method for pooled specificity (*P* < 0.05, see Table 4). Empirically, we also performed an additional subgroup analysis to see if those sequences including DWI and hepatocellular phase enhancement images performed superiorly in CRLM detection. The result was displayed in Fig. 6. The six studies (Fig. 6b) with both DWI and DCE sequences (only included studies which used hepatophilic contrast media as contrast agent) performed higher in the sensitivity (0.94 vs 0.85), but lower in the specificity (0.88 vs 0.89). However, none of these differences was statistically significant (*P* > 0.05 for both sensitivity and specificity). Meanwhile, the performance using liver-specific contrast media (LSCM, such as Evoist) also was compared with those using non-LSCM. As shown in Fig. 7. The ten studies (Fig. 7b) with LSCM performed higher in the sensitivity (0.94 vs 0.83), but lower in the specificity (0.87 vs 0.94). Both of these differences were statistically significant (both *P* > 0.05).

**Table 3:** The result of meta-regression analysis.

covariates		sensitivity				specificity		
	estimate (95%CI)	coefficient	*Z*	*P*	estimate (95%CI)	coefficient	*Z*	*P*
Study design	0.94 [0.86–0.98]	2.80	0.99	0.32	0.92 [0.83–0.96]	2.44	0.67	0.51
Reference standard	0.91 [0.80–0.96]	2.27	−0.25	0.80	0.91 [0.83–0.95]	2.29	0.55	0.58
Field strength	0.89 [0.78–0.94]	2.06	−1.53	0.13	0.89 [0.81–0.94]	2.08	−0.67	0.50
Scanning sequences	0.88 [0.72–0.96]	2.02	−0.75	0.45	0.92 [0.82–0.97]	2.47	0.71	0.48
Imaging planes	0.80 [0.50–0.94]	1.40	−1.66	0.10*	0.82 [0.58–0.94]	1.50	−1.14	0.25
Minimum slice thickness	0.85 [0.66–0.95]	1.75	−0.81	0.42	0.87 [0.71–0.95]	1.93	−0.14	0.89
Type of contrast agent	0.73 [0.33–0.94]	0.99	−1.93	0.05*	0.90 [0.69–0.98]	2.24	−0.02	0.98
Lesion size	0.96 [0.82–0.99]	3.06	0.58	0.56	0.91 [0.82–0.96]	2.29	−0.72	0.47
Publication year#	0.92 [0.83–0.96]	2.43	0.41	0.68	0.89 [0.81–0.94]	2.11	−0.57	0.57
Region	0.88 [0.77–0.95]	2.04	−1.40	0.16	0.87 [0.78–0.92]	1.89	−1.64	0.10*
Enhancement or not	0.93 [0.86–0.96]	2.52	1.70	0.09*	0.91 [0.84–0.95]	2.27	0.95	0.34
Interpretation method	0.88 [0.73–0.95]	1.98	−1.03	0.30	0.83 [0.72–0.90]	1.56	−2.69	0.01*
Patient enrollment	0.87 [0.70–0.95]	1.89	−1.11	0.27	0.92 [0.84–0.96]	2.46	0.89	0.38

#Publication year was binary-coded according to whether the studies were published before the year 2010 or not.

*P value satisfies the pre-set test standard of *α* = 0.10.

**Table 4:** The result of subgroup analysis.

Covariates	Subgroups	No.of studies	Pooled sensitivity (95%CI)	*P* Value	Pooled specificity (95%CI)	*P* Value
No. of imaging planes	≥2	12	0.941 [0.874–0.973]	0.128	0.912 [0.835–0.956]	0.232
	1	4	0.813 [0.616–0.921]		0.861 [0.656–0.952]	
Region	Asian	4	0.970 [0.796–0.996]	0.265	0.949 [0.854–0.983]	0.336
	Europen or American	13	0.881 [0.788–0.937]		0.869 [0.785–0.923]	
Methods	QL	10	0.871 [0.748–0.939]	0.548	0.827 [0.716–0.901]	0.014*
	QT/SemiQT	7	0.941 [0.829–0.982]		0.946 [0.891–0.975]	
Enhancement	With enhancement	15	0.927 [0.852–0.966]	NA	0.905 [0.842–0.944]	NA
Type of contrast agent	Gd-related	11	0.939 [0.865–0.974]	NA	0.899 [0.832–0.941]	NA

*The *P* value smaller than 0.05; Gd: Gadolinium; NA: not applicable; QL: qualitative; QT: quantitative.

## Discussion

Accurate detection of CRLM is vital for CRC patients due to the predictive significance for treatment and survival^33^. The prognosis of CRLM is largely dependent on the resectability of hepatic metastases^34^. Although preoperative CT scan is the first-line imaging modality for metastatic liver lesions, this modality could result in either missed metastases or unnecessary operations^33^. MRI is becoming the current standard in liver metastasis detection since it displayed superior sensitivity to CT with no potential radiation hazard^35–38^. For example, in Floriani *et al*. study^37^, the sensitivity for MRI and CT in the detection of CRLM on a per-lesion basis were 86.3% and 82.6%, respectively, significantly favoring MRI by the calculated odds ratio (0.66). However, opponent evidence also existed among their findings. CT during arterioportography (CTAP) was comparable with MRI either with extra-cellular contrast media or liver-specific contrast media in terms of performance for CRLM detection, though this finding might be unstable because of a small sample size. A more recent study by Choi^35^ further confirmed the superiority of MRI over CT in CRLM detection in both sensitivity and specificity (93.1% vs 82.1% for sensitivity, 87.3% vs 73.5% for specificity). And this conception was also supported by another meta-analysis by Vreugdenburg *et al*.^38^ which showed contrast-enhanced MRI had a higher sensitivity in detecting CRLM than contrast-enhanced CT on either a per-lesion (odds ratio = 1.29, *P* < 0.001) and per-patient basis (odds ratio = 1.21, *P* = 0.010). Based on the current evidences and the fact of having no ionizing radiation, MRI should be recommended as the first line screening scheme in the condition of economically affordable.

One of the advantages of MRI is the availability of multiple scan sequences. Currently, diffusion-weighted magnetic resonance images (DWI) and hepatobiliary phase images with CAs such as gadoxetic acid are among the most sensitive sequences in liver lesion detection. In this meta-analysis, we evaluated the diagnostic performance of contemporary MRI for the detection of hepatic metastasis in patients with CRC. It was also the first meta-analysis, to the best of our knowledge, to make a comprehensive performance profile of MRI in the diagnosis of CRLM.

Despite the superiority to CT, the accuracy of MRI for detecting LM in CRC patients has been controversial according to the literature, and MRI has also displayed a higher FP rate than PET/CT in a recent meta-analysis with gadoxetate disodium-enhanced MRI^35^. In this meta-analysis, we used DOR, PLR, and NLR as our measures of diagnostic accuracy. Generally, PLR greater than 10.0 and NLR less than 0.1 indicate a good diagnostic test. The DOR is the ratio of the overall true judgments relative to the overall false judgments and ranges from 0 to infinity. A higher DOR value indicates higher accuracy. Our results showed that DOR, PLR, and NLR were moderate (62.19, 7.21, and 0.12, respectively). For the sensitivity and specificity, our results showed that the pooled per-lesion sensitivity and specificity of the 17 included studies were 0.90 (95% CI: 0.81–0.95) and 0.88 (95% CI: 0.80–0.92), respectively, with the AUC of the SROC curve of 0.94 (95% CI: 0.92–0.96). The pooled sensitivity of the current study was higher than that in a previous meta-analysis with patients not previously treated (0.80, 95% CI: 0.75–0.85)^36^ or with patients after neoadjuvant chemotherapy (0.86, 95% CI: 0.70–0.94)^39^ and was comparable to that in most recent meta-analyses, which have focused on more advanced sequences such as DWI and contrast-enhanced MRI^35,40^. The results indicated that MRI could be used as a reliable screening tool in clinical practice for CRC patients with a suspicion of LM. However, we also observed notable heterogeneity in the homogeneity test for both sensitivity and specificity. Therefore, considering the moderate measurements of DOR, PLR, NLR, and the substantial heterogeneity, the MRI screening results should be interpreted cautiously as a whole, and the source of heterogeneity needs to be explored to understand the potential factors that may influence the pooled diagnostic performance^41,42^.

Using meta-regression analysis with *α* = 0.10, the factors that may be contributors to the heterogeneity were the number of imaging planes, type of CA, region, whether contrast enhancement was used, and imaging interpretation method. Specifically, studies that used two or more planes, originated in Asia, and used quantitative/semiquantitative methods showed greater sensitivity and specificity than those that used only one imaging plane, originated in Europe or America, and used qualitative methods. However, the subgroup difference was only statistically significant for the imaging interpretation method in specificity (Table 4). It was unexpected that lesion size did not affect the diagnostic performance of MRI in detecting CRLMs. Contemporary MRI has deficits in the detection of small metastatic liver lesions, especially those smaller than 3 mm^8^. In a previous meta-analysis^43^, the accuracy of MRI in detecting CRLM between lesions smaller and larger than 10 mm was significantly different. We noted that one study in our meta-analysis included only lesions larger than 10 mm and had a moderate sensitivity (0.72, 95% CI: 0.59–0.83) and specificity (0.79, 95% CI: 0.54–0.94)^23^. Given that only a small portion of the included studies provided a separate dataset by lesion size, the detection difference according to lesion size could be easily masked. The results also showed that the sequences with DWI did not display superiority over sequences without DWI in the detection of CRLM. This failure to find a significant difference may be attributed to the factor that most of the included studies adopted contrast-enhanced sequences, either with Gd-related CA or SPIO, since DWI and contrast-enhanced imaging have comparable sensitivities in the detection of CRLM^40^. Although there was no publication bias found in the included studies using Deek’s funnel plot, the publication language was limited in English in this meta-analysis, which could have introduced a potential bias.

For the subgroup analyses, though there was no significant difference between the performance of those combined LSCM enhancement with DWI and of those not, our results did reveal a trend that the scanning protocol which included both diffusion-weighted and hepatocellular phase images performed over those not (94% vs 85% for sensitivity). The hepatocyte-deficient tumors such as CRLM normally had lower signal intensity in a higher intensity liver background during the hepatobiliary phase, thus making the tumors conspicuous in liver parenchyma and leading to elevated sensitivity^24,44^. Our result conservatively supported this point. The limited study sample size may decrease the statistical power for a significant difference to manifest. We also performed a comparison between LSCM enhancement like Evosit and those using conventional CAs for the detection of CRLM. Though no significant difference was found again, the results tended to tell that LSCM, comparing with conventional CAs, did increase the true positive rate at the cost of increasing the false positive rate either. In a previous meta-analysis in which the results favored MRI^37^, the difference between MRI and CT in detecting CRLM was higher when LSCM were administered than when conventional CAs were used. This could be an indirect evidence to support the superiority of LSCM. However, no direct and robust evidence was established yet. And based on our results, we conservatively recommended the use of LSCM in the diagnosis of suspicious CRLM.

Additionally, our results showed that MRI detection accuracy for CRLM was greater in studies from Asia than in those from Europe or America, although this difference was statistically nonsignificant. It is unclear why the result favored studies from Asia, however, the four studies from Asia were almost all the most recently published papers (one in 2015^9^, one in 2017^31^, and the other two in 2018^10,28^). Although our results did not show a year-related trend when the studies before and after 2010 were compared, a previous meta-analysis did indicate that MRI sensitivity in the detection of CRLM in studies after 2004 was significantly increased compared with those before 2004^36^. Our result also favored studies that used two or more imaging planes rather than those that used only one plane. This result is consistent with that of a recent study focusing on the diagnostic performance of MRI in bone metastases from prostate cancer^45^. Another finding was the better performance of the quantitative/semiquantitative method for imaging interpretation than the qualitative method, especially for specificity, indicating that a quantitative/semiquantitative method used for the interpretation of imaging may reduce the FP rate.

Some limitations of this meta-analysis should be acknowledged. First, per-patient-based analyses were not performed in our study because of the limited data on a per-patient basis. Although a per-lesion-based analysis could be more accurate and could provide crucial information, such as lesion size, number and location, which are required for developing a therapeutic strategy, it is still important to differentiate patients with metastatic lesions from those without metastatic lesions. Second, although there was no significant publication bias in this study, selective reporting biases could exist since reviews, conference abstracts and letters to the editors as well as data published in languages other than in English were excluded. Moreover, the power of the funnel plots might have been low due to the limited sample size of the included studies in this meta-analysis. Third, notable heterogeneity was observed among the included studies. Although we investigated possible sources of heterogeneity by meta-regression analysis, the exploration of heterogeneity may still have been inadequate since the variables collected from the included studies were limited. Additionally, comparisons between some of the subgroups were unavailable because of the limited sample size. Finally, a majority of the included studies were retrospectively designed and used multiple reference standards, which can be considered limitations and potentially bias the results.

## Conclusions

In conclusion, our meta-analysis shows that MRI demonstrated high sensitivity and specificity for the detection of LM from CRC. Studies using multiple imaging planes for the assessment showed higher diagnostic accuracy than those using only one plane. Imaging interpretation with quantitative/semiquantitative methods was superior to qualitative methods.

Advanced techniques such as scanning with DWI and liver-specific CAs tends to be more sensitive. Nonetheless, given the notable heterogeneity and inherent limitations, large-scale, prospectively designed trials are needed to verify the clinical value of MRI, especially for the added value of DWI and liver-specific CAs enhanced MR imaging.
